# Finite-Temperature Correlation Functions Obtained from Combined Real- and Imaginary-Time Propagation of Variational Thawed Gaussian Wavepackets

**DOI:** 10.3390/e26050412

**Published:** 2024-05-10

**Authors:** Jens Aage Poulsen, Gunnar Nyman

**Affiliations:** Department of Chemistry and Molecular Biology, University of Gothenburg, SE 413 90 Gothenburg, Sweden

**Keywords:** tunneling, variational principle, wavepacket, boltzmann distribution

## Abstract

We apply the so-called variational Gaussian wavepacket approximation (VGA) for conducting both real- and imaginary-time dynamics to calculate thermal correlation functions. By considering strongly anharmonic systems, such as a quartic potential and a double-well potential at high and low temperatures, it is shown that this method is partially able to account for tunneling. This is contrary to other popular many-body methods, such as ring polymer molecular dynamics and the classical Wigner method, which fail in this respect. It is a historical peculiarity that no one has considered the VGA method for representing *both* the Boltzmann operator and the real-time propagation. This method should be well suited for molecular systems containing many atoms.

## 1. Introduction

The idea of using Gaussian wavepackets with flexible widths for approximating the time evolution of wavefunctions was first proposed by Heller [1]. The dynamics of these wavepackets, called thawed Gaussian wavepackets, are exact for multi-dimensional harmonic problems and are thus well suited for molecular problems that involve vibrational quantum effects [2,3,4]. An elaboration of the method was later put forth by Metiu [5] as well as Coalson and Karplus [3] who applied the variational principle of McLachlan to minimize the error between approximate and exact thawed Gaussian time propagation. The wavepackets obtained from this variational Gaussian approximation (VGA) still have the same form as those by Heller, but they now move on an *effective* potential. A key consequence of this is the ability of the VGA method to partially describe tunneling [2,6].

Today, different Gaussian wavepackets, including their frozen counterparts, enjoy immense popularity, due to their conceptual and computational simplicity. They serve as moving basis functions in many advanced computational schemes that couple them together to enable the study of tunneling and non-adiabatic effects in complex molecular systems. Among these, we mention coupled coherent states [7], the multiple spawning method by Ben-Nun and Martinez [8] and the variational multi-configurational Gaussian (vMCG) method [9], all of which employ Heller’s Gaussian wavepackets in one form or another. The variational Gaussian wavefunction approximation also comes in handy when computing the Boltzmann operator, which is equivalent to a propagation of wavefunctions in imaginary time. Indeed, Frantsuzov and Mandelshtam [10] published the equations of motion for a variational Gaussian wavepacket moving in imaginary time, and the temperature-dependent structure of atomic Lennard–Jones clusters was successfully calculated. This imaginary-time version of the VGA principle is computationally very effective and has been adopted by Miller and Liu for representing the Boltzmann operator for complex systems such as liquids [11,12]. We notice that Cartarius and Pollak [13] have proposed a frozen Gaussian version of Mandelshtam’s VGA method.

In the last two decades, new schemes have been developed aimed at simulating the temperature-dependent real-time dynamics of complex quantum systems. More specifically, these methods target the calculation of correlation functions, which is the natural object of interest when computing reaction rate constants, diffusion constants, Van Hove spectra, etc. Among the methods, we mention ring polymer molecular dynamics (RPMD) [14,15], path integral Liouville dynamics (PILD) [16], linearized semi-classical initial value representation (LSC-IVR) [17] and the equivalent so-called classical Wigner (CW) approach [18,19]. We mention that LSC-IVR and CW utilize a sampling of quantum initial conditions through a Boltzmann–Wigner transform, while the dynamics are approximated by classical trajectories. RPMD, on the other hand, adopts an effective quantum potential through a Feynman ring polymer necklace. These methods all have their origin in statistical mechanics through Feynman path integrals and are not based on wavefunctions. They easily handle hundreds of degrees of freedom but are, on the other hand, not capable of describing genuine coherence effects. Common to all of them is the requirement to represent the Boltzmann operator in a computationally manageable form. As mentioned, Liu and Miller have adopted Mandelshtam’s VGA for this purpose [11,20].

The VGA has been applied by Buch [2] to simulate the real-time dynamics of neon clusters. Also, Vaníček and Fereidan [6] recently applied VGA to study the real-time quantum dynamics of twenty coupled Morse oscillators. Neither study employed a Boltzmann sampling. To our knowledge, no work has considered the combined usage of VGA for both sampling the Boltzmann statistics and performing the real-time dynamics for any problem (we note that Frantsuzov and Mandelshtam mentioned this as a possibility [10], although the idea was never pursued). If this can be performed for large systems, then the VGA method could potentially compete with the RPMD and CW methods. Two questions appear in this connection: the first is the level of accuracy of such a combined VGA method, while the second is its ease and practicality of implementation. We will focus on the first question here. In particular, since the VGA, at least in principle, can handle large dimensional systems, we compare it to other multi-dimensional methods, such as RPMD and the classical Wigner model. In this paper, we focus on challenging one-dimensional model problems.

## 2. Methods

We will test the various methods by calculating the symmetrized position correlation function (CF) given by

$$G\left(t\right)=\frac{1}{Z}Tr\left\{\hat{x}\left(t\right)\exp\left(-\frac{\beta }{2}\hat{H}\right)\hat{x}\exp\left(-\frac{\beta }{2}\hat{H}\right)\right\}$$

(1) $$=\frac{1}{Z}Tr\left\{\exp\left(i\hat{H}t/\hbar \right)\hat{x}\exp\left(-i\hat{H}t/\hbar \right)\exp\left(-\frac{\beta }{2}\hat{H}\right)\hat{x}\exp\left(-\frac{\beta }{2}\hat{H}\right)\right\}.$$

where *Z* denotes the trace of the Boltzmann operator: $Z=Tr\{\exp\left(-\beta \hat{H}\right)\}$. Focusing first on the VGA approach, we calculate its approximation to G(t) as follows: First, we utilize the VGA to the Boltzmann operator as proposed by Frantsuzov and Mandelshtam [10]:

(2) $$\exp\left(-\frac{\beta }{2}\hat{H}\right)|x_{0}\rangle ≃|g(x_{0};\frac{\beta }{2})\rangle ,$$

where the so-called thermal Gaussian wavefunction is given by

(3) $$\langle x\left|g(x_{0};\frac{\beta }{2})\right.\rangle =\exp\left(-\frac{1}{2}\Lambda \left(\frac{\beta }{2},x_{0}\right)(x-x_{0}\left(\frac{\beta }{2}\right){)}^{2}+\gamma \left(\frac{\beta }{2},x_{0}\right)\right).$$

Equation (2) tells us that the thermal wavepacket is the broadening of the delta function $|x_{0}\rangle$ when propagated an amount of $\frac{\beta \hbar }{2}$ in imaginary time. Thermal wavepackets appear mathematically identical to Gaussian wavepackets but differ in one important way: the thermal Gaussians are not normalized to unity but instead have a very small amplitude at low temperature (a large negative value of $\gamma (\frac{\beta }{2},x_{0})$). For brevity, we suppress the $x_{0}$-dependence of width Λ and “phase-factor” γ in the remainder of this text. All parameters $\Lambda (\frac{\beta }{2})$, $x_{0}\left(\frac{\beta }{2}\right)$ and $\gamma (\frac{\beta }{2})$ are propagated in imaginary time from τ=0 to τ=βℏ/2. We refer to [10] for details. By using closure and utilizing Equation (2), we may write

$$\exp\left(-\frac{\beta }{2}\hat{H}\right)\hat{x}\exp\left(-\frac{\beta }{2}\hat{H}\right)=\int dx_{0}x_{0}\exp\left(-\frac{\beta }{2}\hat{H}\right)|x_{0}\rangle \langle x_{0}|\exp\left(-\frac{\beta }{2}\hat{H}\right)$$

(4) $$≃\int dx_{0}x_{0}|g(x_{0};\frac{\beta }{2})\rangle \langle g(x_{0};\frac{\beta }{2})|.$$

Equation (4) is very convenient, since it expresses the Boltzmann operator in a diagonal basis of thermal Gaussians. By inserting Equation (4) into Equation (1), we obtain

(5) $$G\left(t\right)=\frac{1}{Z}\int dx_{0}x_{0}Tr\left\{\hat{x}\exp\left(-i\hat{H}t/\hbar \right)|g(x_{0};\frac{\beta }{2})\rangle \langle g(x_{0};\frac{\beta }{2})|\exp\left(i\hat{H}t/\hbar \right)\right\}.$$

It follows from Equation (5) that the partition function is now calculated as

(6) $$Z=\int dx_{0}Tr\left\{|g(x_{0};\frac{\beta }{2})\rangle \langle g(x_{0};\frac{\beta }{2})|\right\}=\int dx_{0}\langle g(x_{0};\frac{\beta }{2})|g(x_{0};\frac{\beta }{2})\rangle .$$

As Equation (5) shows, a real-time propagation of the thermal wavepackets is required. To do so, we can use the variational principle of McLachlan [21] applied to the wavepacket:

(7) $$\langle x\left|g(x_{0},p_{0};t)\right.\rangle =\exp\left(\frac{1}{2}\frac{i}{\hbar }D\left(t\right){\left(x-x_{0}\left(t\right)\right)}^{2}+\frac{i}{\hbar }p_{0}\left(t\right)\left(x-x_{0}\left(t\right)\right)+\frac{i}{\hbar }w\left(t\right)\right).$$

where D(t) and w(t) are complex parameters, while $x_{0}\left(t\right)$ and $p_{0}\left(t\right)$ are real. Clearly, this wavepacket has the same form as Equation (3). The VGA equations of motion for $x_{0}\left(t\right)$, $p_{0}\left(t\right)$, D(t) and w(t) can be found in Refs. [2,3] and are also given in Appendix A. One may alternatively perform the VGA time dynamics of the wavepacket in Wigner phase space. Indeed, as shown in Ref. [22], as an alternative to the McLachlan variational principle, a more general, time-dependent “action principle” can be derived for a general trial Wigner function $O_{W}^{\vec{\alpha }}$ that depends on a time-dependent parameter vector $\vec{\alpha }$. This variational principle claims the stationarity of a functional *J*:

(8) δJ=0,

with

$$J\left[\vec{\alpha }\right]=\int_{0}^{t}ds\int dqdp(O_{W}^{\vec{\alpha }}\left[q,p\right]{)}^{*}\times$$

(9) $$\left(\hat{L}-\dot{\vec{\alpha }}\nabla_{\vec{\alpha }}\right)\left(O_{W}^{\vec{\alpha }}\left[q,p\right]\right),$$

where δ denotes *complex* variations in the time derivative of $\vec{\alpha }$. $\hat{L}$ is the Liouvillian. By following Ref. [23], we apply Equation (9) to a general Gaussian Wigner function (not necessarily a minimum uncertainty wavefunction but a truly mixed state) of the form

(10) $$W_{G}\left(q,p;t\right)=N\exp\left(-{\left(\begin{array}{c} q-q_{0}\left(t\right)\\ p-p_{0}\left(t\right) \end{array}\right)}^{T}\left(\begin{array}{cc} \alpha (t) & \theta (t)\\ \theta (t) & \lambda (t) \end{array}\right)\left(\begin{array}{c} q-q_{0}\left(t\right)\\ p-p_{0}\left(t\right) \end{array}\right)\right)$$

with parameters $\left(q_{0}\left(t\right),p_{0}\left(t\right),\alpha \left(t\right),\theta \left(t\right),\lambda \left(t\right)\right)$. We then obtain the following equations of motion [23]:

(11) $$\frac{d}{dt}\left(\begin{array}{c} q_{0}\\ p_{0}\\ \alpha \\ \theta \\ \lambda \end{array}\right)=\left(\begin{array}{c} p_{0}/M\\ -\frac{d}{dq}V_{sm}\left(q_{0}\right)\\ 2\theta \times M\Omega^{2}\left(q_{0}\right)\\ \lambda \times M\Omega^{2}\left(q_{0}\right)-\frac{\alpha }{M}\\ -2\frac{\theta }{M} \end{array}\right),$$

where the effective frequency $\Omega (q_{0})$ is defined by

(12) $$M\Omega^{2}\left(q_{0}\right)=\frac{d^{2}}{dq_{0}^{2}}V_{sm}\left(q_{0}\right).$$

The smeared potential is

(13) $$V_{sm}\left(q_{0}\right)=\frac{1}{\sqrt{\pi \hbar^{2}\lambda^{\prime }}}\int_{-\infty }^{+\infty }dyV\left(y\right)\exp\left(-\frac{{\left(q_{0}-y\right)}^{2}}{\lambda^{\prime }\hbar^{2}}\right),$$

with

(14) $$\lambda^{\prime }\left(t\right)=\frac{\lambda }{2}\left\{1+\frac{1}{\hbar^{2}\left(\alpha \lambda -\theta^{2}\right)}\right\}.$$

where $\alpha \lambda -\theta^{2}$ is a constant of motion [23] and so is the energy of the Wigner function. Since we restrict ourselves to the propagation of a minimum uncertainty wavepacket, we have $\alpha \lambda -\theta^{2}=1/\hbar^{2}$ and, consequently, $\lambda^{\prime }\left(t\right)=\lambda$ [23]. We emphasize again that these equations give identical results as a direct variational propagation of the coherent state using the variational principle of McLachlan. The above equations will, therefore, also be referred to as VGA equations.

As shown in Appendix B, by combining Equations (5) and (10), we obtain

(15) $$G\left(t\right)=\frac{1}{Z}\int dx_{0}x_{0}q_{0}\left(t\right)\exp\left(2\gamma \left(\frac{\beta }{2}\right)\right)\sqrt{\frac{\pi }{\Lambda (\frac{\beta }{2})}},$$

where $q_{0}\left(t\right)$, $\gamma (\frac{\beta }{2})$ and $\Lambda (\frac{\beta }{2})$ are all understood to be functions of $x_{0}$.

The classical Wigner method may be derived rather easily from Equation (5). First, we introduce the Wigner function of operator $\hat{A}$ as [18]

(16) $${\left(\hat{A}\right)}_{W}\left[q,p\right]=\int_{-\infty }^{\infty }d\eta \exp\left(-ip\eta /\hbar \right)\langle x+\frac{1}{2}\eta \left|\hat{A}\right|x-\frac{1}{2}\eta \rangle ,$$

which, used together with the identity

$$Tr\left\{\hat{A}\hat{B}\right\}=\int \int \frac{dqdp}{2\pi \hbar }{\left(\hat{A}\right)}_{W}\left[q,p\right]{\left(\hat{B}\right)}_{W}\left[q,p\right]$$

transforms Equation (5) into

$$G\left(t\right)=\frac{1}{Z}\int dx_{0}x_{0}Tr\left\{\hat{x}\exp\left(-i\hat{H}t/\hbar \right)|g(x_{0};\frac{\beta }{2})\rangle \langle g(x_{0};\frac{\beta }{2})|\exp\left(i\hat{H}t/\hbar \right)\right\}$$

$$=\frac{1}{Z}\int dx_{0}x_{0}Tr\left\{\exp\left(i\hat{H}t/\hbar \right)\hat{x}\exp\left(-i\hat{H}t/\hbar \right)|g(x_{0};\frac{\beta }{2})\rangle \langle g(x_{0};\frac{\beta }{2})|\right\}$$

$$=\frac{1}{Z}\int dx_{0}x_{0}\int \int \frac{dqdp}{2\pi \hbar }{\left(\exp\left(i\hat{H}t/\hbar \right)\hat{x}\exp\left(-i\hat{H}t/\hbar \right)\right)}_{W}\left[q,p\right]$$

(17) $${\left(|g(x_{0};\frac{\beta }{2})\rangle \langle g(x_{0};\frac{\beta }{2})|\right)}_{W}\left[q,p\right].$$

The classical Wigner model [18] eliminates the time-dependent Heisenberg operator $\exp\left(i\hat{H}t/\hbar \right)\hat{x}\exp\left(-i\hat{H}t/\hbar \right)$ by moving its time dependence into the phase space argument of the Wigner functions using classically propagated trajectories $\left(q_{t},p_{t}\right)$:

$$G\left(t\right)≃\frac{1}{Z}\int dx_{0}x_{0}\int \int \frac{dqdp}{2\pi \hbar }q\left(q_{t},p_{t}\right){\left(|g(x_{0};\frac{\beta }{2})\rangle \langle g(x_{0};\frac{\beta }{2})|\right)}_{W}\left[q,p\right]$$

(18) $$=\frac{1}{Z}\int dx_{0}x_{0}\int \int \frac{dqdp}{2\pi \hbar }q_{t}{\left(|g(x_{0};\frac{\beta }{2})\rangle \langle g(x_{0};\frac{\beta }{2})|\right)}_{W}\left[q,p\right].$$

G(t) computed in this way will be exact up to and including quadratic potentials and for high temperatures.

Finally, we consider the RPMD methodology for computing G(t). RPMD cannot directly calculate G(t), but instead, it calculates its analogous Kubo-transformed correlation function [14,15]:

(19) $$C_{K}\left(t\right)=\frac{1}{\beta \hbar Z}\int_{0}^{\beta \hbar }d\lambda Tr\left\{\exp\left(i\hat{H}t/\hbar \right)\hat{x}\exp\left(-i\hat{H}t/\hbar \right)\exp\left(-\left(\beta -\lambda \right)H\right)\hat{x}\exp\left(-\lambda \hat{H}\right)\right\}.$$

It does so by considering classical dynamics in an extended *n* dimensional phase space defined by the classical Hamiltonian [15]

(20) $$H_{n}\left(\vec{x},\vec{p}\right)=\sum_{i=1}^{n}\frac{p_{i}^{2}}{2m}+\frac{m}{2\beta_{n}^{2}\hbar^{2}}\sum_{i=1}^{n}{\left(x_{i}-x_{i-1}\right)}^{2}+\sum_{i=1}^{n}V\left(x_{i}\right),$$

with $\beta_{n}=\beta /n$. The harmonic spring terms hold $\left\{x_{i}\right\}$ together in a necklace and its centroid $\bar{x}$ defined as

(21) $$\bar{x}=\frac{1}{n}\sum_{i=1}^{n}x_{i}.$$

The centroid is used to compute the RPMD approximation to $C_{K}\left(t\right)$ by using [15]

(22) $$C_{K}\left(t\right)≃\frac{1}{{\left(2\pi \hbar \right)}^{n}Z_{n}}\int d\vec{x}\int d\vec{p}\exp\left(-\beta_{n}H_{n}\left(\vec{x},\vec{p}\right)\right)\bar{x}\left(0\right)\bar{x}\left(t\right),$$

with

(23) $$Z_{n}=\frac{1}{{\left(2\pi \hbar \right)}^{n}}\int d\vec{x}\int d\vec{p}\exp\left(-\beta_{n}H_{n}\left(\vec{x},\vec{p}\right)\right).$$

Once $C_{K}\left(t\right)$ is obtained, a convolution of it turns it into G(t); see Appendix C.

## 3. Results

**Figure 1:**
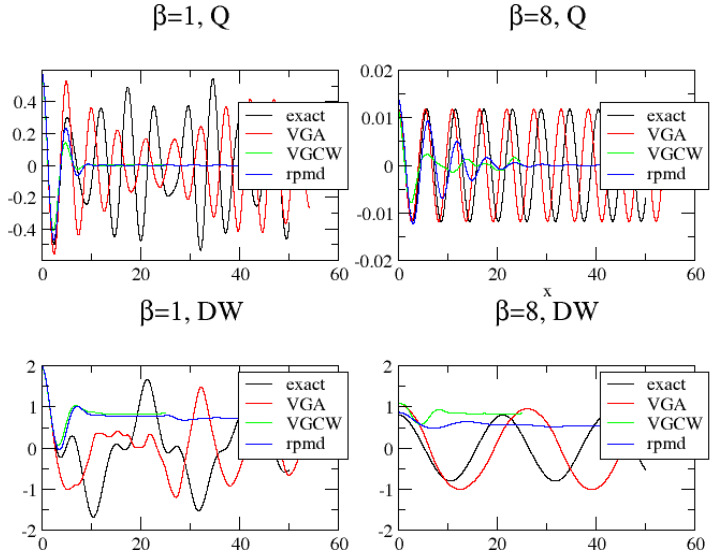
The position correlation function G(t) as calculated for a quartic (*Q*) and a double-well potential (DW) for β={1,8}.

We apply the VGA approximation in Equation (15) for calculating G(t) for two strongly anharmonic potentials often considered in the literature; see, e.g., [24] and references therein. These are defined by M=1, ℏ=1 and $k_{B}=1$. We set $V\left(x\right)=-\frac{1}{2}x^{2}+x^{4}/10$ or $V\left(x\right)=x^{4}/4$. These two potentials are referred to as the double-well (DW) and quartic potential (*Q*), respectively, and both are symmetric around x=0. We also use the analytic Wigner transform of Equation (4) to generate initial conditions for the so-called classical Wigner model. This method is called variational Gaussian Classical Wigner (VGCW) model. Finally, we also compute G(t) from its Kubo-transformed analogue, $C_{K}\left(t\right)$, obtained by RPMD. As shown in Appendix C, it is possible to obtain G(t) by performing a simple convolution of $C_{K}\left(t\right)$. In Figure 1, we show the results of these methods when applied to the two potentials at low (β=8) and high (β=1) temperature.

It is clearly seen that VGA outperforms the other approximate methods at low temperature for both potentials. Specifically, the VGA clearly tunnels through the barrier at β=8, while the other methods fail completely for the DW potential. Also, for the quartic potential at low temperature, the VGA method dephases much slower than the other methods. A proper definition of tunneling would be in its place. We adopt the simple definition that a particle tunnels in the DW potential if G(t) changes sign during its time evolution.

At high temperature, the RPMD and VGCW correlation functions approach each other. However, the VGA result does *not* converge to the RPMD or VGCW correlation function but instead remains non-classical in its dynamics. It is difficult to say if the RPMD and VGCW results are better than the VGA results or vice versa. The seemingly non-classical behavior of VGA at high temperature may be explained as follows: At high *T*, the VGA wavefunction is sharply peaked around $x_{0}$. This means that α starts out being big too when performing the time propagation of the Wigner function. From the equations of motion shown in Equation (11), we see that θ, therefore, becomes negative, which in turn leads to a large positive value of λ. Finally, a large value of λ leads to a large non-classical potential smearing, as seen from Equations (13) and (14). We conclude that this non-classical limit must be an inherent consequence of using the optimal smeared potential as prescribed by the variational principle *together* with an initial sharply peaked wavefunction.

**Figure 2:**
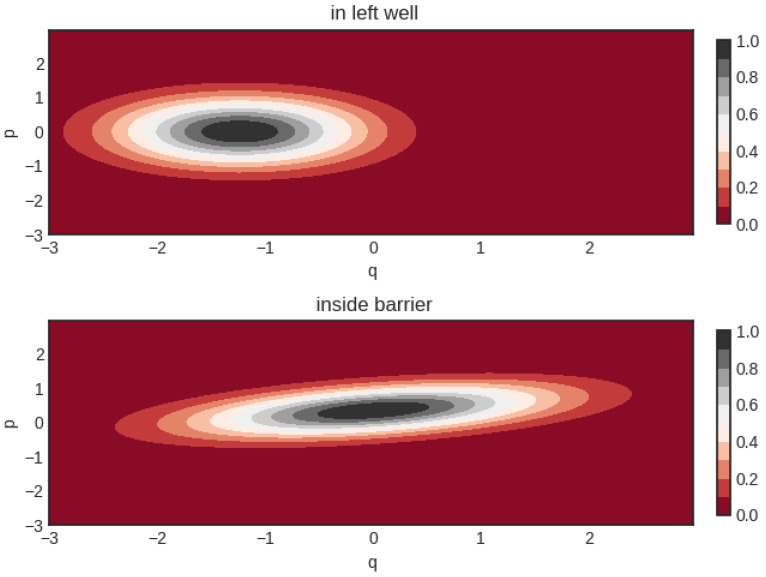
Shape of the Gaussian Wigner function when tunneling. Initial parameters of Wigner function are $q_{0}=-1.237$, $p_{0}=\theta =0$ and α=1/λ=0.864.

The ability of the VGA method to tunnel can perhaps be best explained by considering the time evolution of its Wigner function. In Figure 2, we show the shape of the Wigner function for a tunneling case in the double well very similar to the low temperature case shown in Figure 1. Inside the barrier, the Wigner function reduces its potential energy by stretching out along the *q*-direction (changing to small α) while simultaneously compressing along *p* (adopting a large λ). A quick inspection of Equation (11) shows that a change in α and λ is only possible for a non-zero value of θ (which corresponds to a non-zero real part of the wavefunction width, D(t)). Without the cross terms $\theta \left(p-p_{0}\left(t\right)\right)\left(q-q_{0}\left(t\right)\right)$ in the Wigner function ansatz, there will be no tunneling.

**Figure 3:**
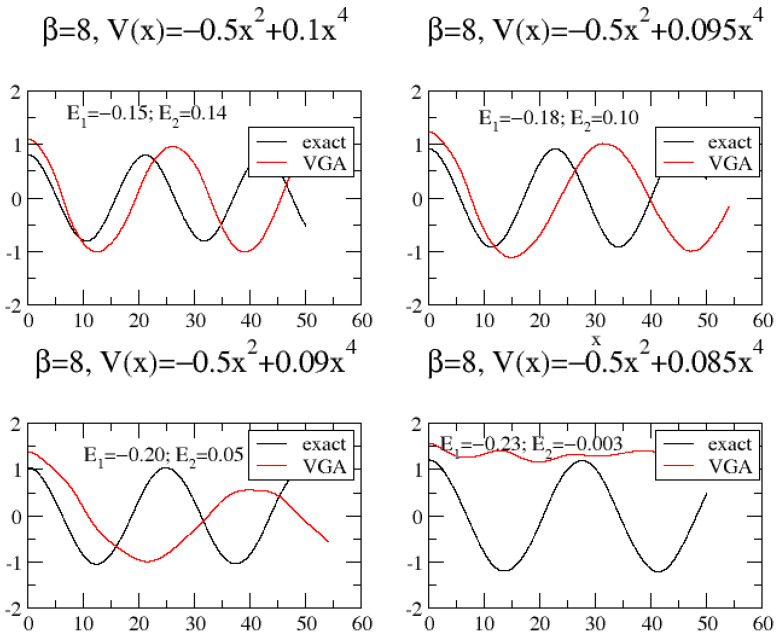
VGA and exact results for G(t) for slightly different double-well potentials.

**Figure 4:**
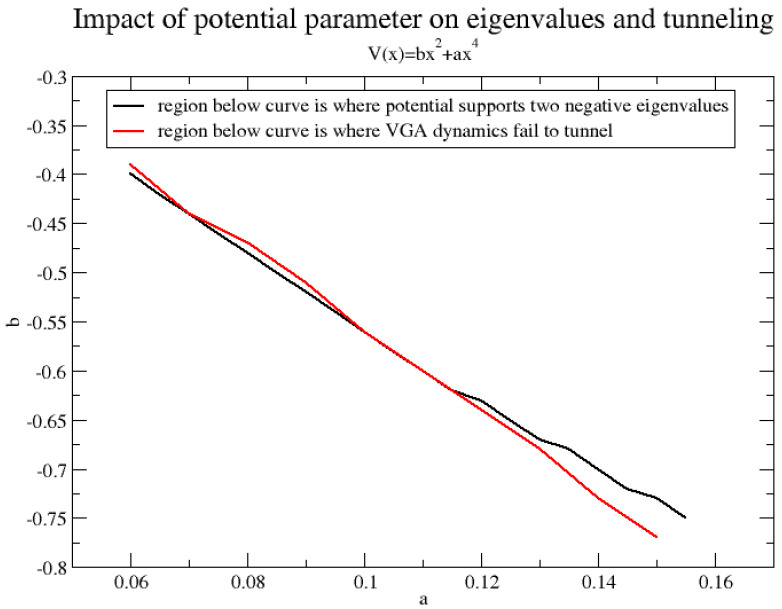
Domains of (i) two or more negative eigenvalues/maximum one negative eigenvalue and (ii) tunneling/not tunneling for the VGA method.

The ability of the VGA method to treat tunneling of course depends on the potential. In fact, by varying the DW potential parameter, *a*, in $V\left(x\right)=bx^{2}+ax^{4}$, one easily encounters cases where tunneling is incorrectly absent in the VGA result. This is shown in Figure 3, where a=0.085 apparently makes the barrier too wide for VGA to result in tunneling. We mention that the energy of the wavepacket is *not* a good indicator of successful tunneling. In fact, for a=0.085, the wavepacket energy is slightly higher than the potential energy at the barrier top. In Figure 3, we show the values of the two lowest eigenvalues. It appears that VGA *can only tunnel whenever only one eigenvalue is below zero*, the potential energy value at the barrier top. That this criterion makes sense also more generally can be observed by performing the following experiment, which checks the variational equations in Equation (11) for their ability to model tunneling: Place a wavepacket (or Wigner function) (where the wavepacket is obtained by first using the standard variational principle to minimize the energy of

$$\Psi =N\{\exp\left(-\frac{\alpha }{2}{\left(x-x_{0}\right)}^{2}\right)+\exp\left(-\frac{\alpha }{2}{\left(x+x_{0}\right)}^{2}\right)\};$$

afterwards, we keep, e.g., $\Psi ={\left(\frac{\alpha }{\pi }\right)}^{\frac{1}{4}}\exp\left(-\frac{\alpha }{2}{\left(x-x_{0}\right)}^{2}\right)$ as our coherent state) in one of the wells of the potential $V\left(x\right)=bx^{2}+ax^{4}$ and use the variational principle in Equation (11) to drive it forward in time. If the average position of the wavepacket changes sign, we say that it tunnels. This is then repeated for a large number of combinations of (a,b) values. Also, for each potential, we observe the number of negative eigenvalues. The results are shown in Figure 4, which shows two curves in parameter space (a,b). The first one separates the space into a tunneling and not-tunneling domain for the variational method. The other curve separates the space into a domain in which the potential has at least two negative eigenvalues and one with a maximum of one. We see a good correlation between the curves. However, in the corner of the figure, where the magnitude of both (a,b) is large, it becomes worse. Here, it is possible to *both* have two eigenvalues below zero and make the variational wavepacket tunnel. However, generally speaking, a rough criterion for tunneling is that we have only one eigenvalue below the barrier top.

From Figure 4, we find that the ability of the VGA method to tunnel is, after all, quite limited. Clearly, a not too costly way to improve the tunneling dynamics is desirable. One possibility would be to dynamically couple several variational thawed Gaussians together. There are many possible ways of coupling Gaussian wavefunctions. To only name a few, we mention the coupled coherent states method employing a set of fixed-width Gaussians [7,25]; the fully variationally coupled Gaussian method by Burghardt and coworkers [9]; and the multiple spawning method by Ben-Nun and Martinez [8], which adds (spawns) new frozen Gaussian basis functions in classically forbidden regions, which are formed on the fly. Perhaps, the latter method is the most practical if only a very limited number of Gaussians are to be coupled.

As an illustration, we apply the spawning method for calculating G(t) for the potential $V\left(x\right)=-0.6x^{2}+0.1x^{4}$ at β=8. Contrary to Ben-Nun and Martinez, we do not use frozen Gaussians but adopt our time-dependent VGA Gaussians as basis functions. We perform a very limited calculation by using only eleven VGA functions to represent the Boltzmann operator in Equation (4). We perform separate dynamics of these eleven VGA functions. None of these can tunnel through the barrier; so, according to the spawning algorithm, we should add a basis function in the other well (which is chosen identical to the original function except for $q_{0}\to -q_{0}$). We thus have eleven 2×2 problems, which may be written in standard form by using notation from Ben-Nun and Martinez [8]:

(24) $$\underline{S}\dot{\vec{c}}=\left(\frac{1}{i\hbar }\underline{H}-\underline{\dot{S}}\right)\vec{c},$$

where $\vec{c}$ holds the coefficients for the wavefunction $\Psi =c_{1}g_{1}\left(x_{0},p_{0};t\right)+c_{2}g_{2}\left(x_{0},p_{0};t\right)$. The overlap matrix and the Hamiltonian matrix are

(25) $$S_{ij}=\int_{-\infty }^{\infty }dxg_{i}^{*}\left(x\right)g_{j}\left(x\right),$$

(26) $$H_{ij}=\int_{-\infty }^{\infty }dxg_{i}^{*}\left(x\right)\hat{H}g_{j}\left(x\right).$$

$\underline{\dot{S}}$ is defined as

(27) $${\dot{S}}_{ij}=\int_{-\infty }^{\infty }dxg_{i}^{*}\left(x\right){\dot{g}}_{j}\left(x\right).$$

**Figure 5:**
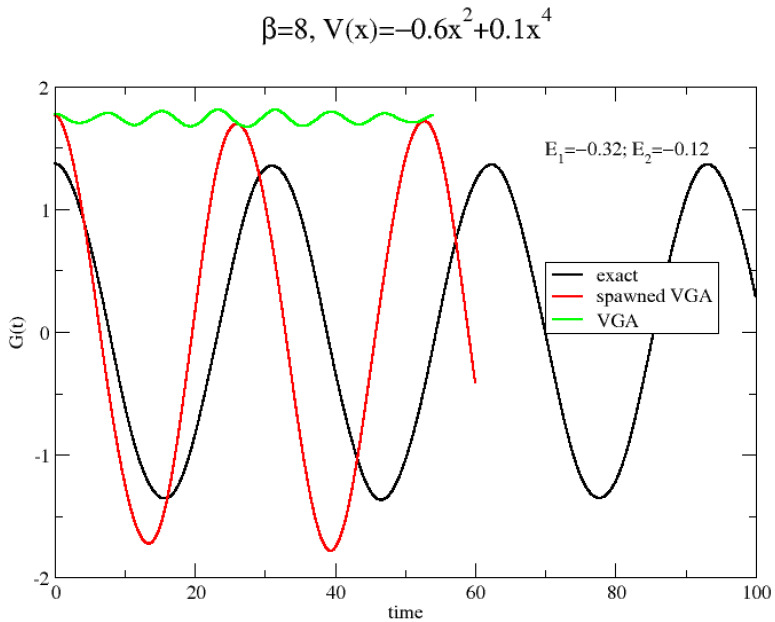
Dynamics for the low-temperature double-well problem. G(t) calculated by both coupled VGA functions (one original and one spawned) and a single VGA function.

In Figure 5, G(t) obtained by adopting this very economic version of the multiple spawning method (in this case, a single spawning method) is shown. Although the result is far from perfect, tunneling is now accounted for.

## 4. Conclusions

The quality of correlation functions derived from the variational Gaussian approximation (VGA) was investigated for two challenging model problems at both high and low temperature. Compared with RPMD and the classical Wigner (CW) method, it was shown that the VGA method is superior at low temperature; it is able to account for tunneling and also has longer coherence time. However, a drawback of the method is that it lacks a well-defined classical high-temperature limit. As a rule of thumb, we further found that the VGA method does not yield tunneling when two quantum states lie below the barrier top. For such a case, a very simple implementation of the multiple spawning method [8] was considered, and the ability of the upgraded method to tunnel was demonstrated.

As mentioned, there is a large number of effective numerical methods available that propagate coupled Gaussian wavepackets. There is no reason as to why the combined thermal and real-time VGA method cannot be combined further with such methods. We should also address the feasibility and practicality of VGA for conducting combined imaginary- and real-time dynamics for large complex systems. The VGA method is a proven method for sampling thermal Gaussians in the canonical ensemble for clusters and liquids [11,20]. The more challenging part is its real-time dynamics. When Buch simulated the real-time dynamics of high-energy liquid-like neon clusters by using the VGA method, she encountered an “unchecked wavepacket broadening in orthogonal directions that represent cluster rotations in the small displacement limit” [2]. More specifically, it was the *real* part of D(t) in Equation (7) that increased in value. Buch also observed a gradual energy transfer from the center of the wave packet motion, to kinetic energy terms originating from the real part of D(t). These stability issues were, however, resolved by periodically resetting the real part of D(t) to zero. Buch did not sample wavepackets from a Boltzmann distribution, but instead, the problematic configuration corresponded to a high-energy trajectory. We should mention that Fereidan and Vaníček [6] implemented VGA to study the real-time dynamics of twenty coupled Morse oscillators. They did not report on any stability problems. It is difficult to say how big this problem would be in general and further studies are clearly desirable. One could for instance try to implement the combined imaginary–real-time VGA dynamical scheme for a simple liquid problem.

We have considered the VGA method for performing combined imaginary- and real-time dynamics. We should summarize which formal rules of quantum dynamics this method obeys. One important question is whether this model is capable of conserving the canonical ensemble from which the initial conditions are sampled. More precisely, would

(28) $$G\left(t\right)=\frac{1}{Z}Tr\left\{\hat{A}\left(t\right)\exp\left(-\frac{\beta }{2}\hat{H}\right)\hat{B}\exp\left(-\frac{\beta }{2}\hat{H}\right)\right\}$$

be time-independent if we set $\hat{B}=\hat{I}$? The answer is negative, as can be checked numerically. This is undoubtedly a shortcoming of the method compared with competing methods, e.g., RPMD [14], the planetary Feynman–Kleinert linearized path integral method (PFK-LPI) [24] and the path integral Liouville dynamics (PILD) method and its various implementations as proposed by Liu [16,26] and Liu and Miller [12,27], which all preserve the canonical ensemble. One could speculate that the problem with the VGA method is that it samples Gaussians with *real* exponents (see Equation (3)) but employs *complex* exponents in the dynamics.

As already discussed, another formal property not fulfilled by the VGA method, as opposed to RPMD, PFK-LPI and PILD, is that it lacks a high-temperature classical limit for anharmonic potentials. However, as opposed to RPMD, the VGA method considered *does* handle non-linear correlation functions, as well as linear ones (a property shared by the CW, PFK-LPI and PILD methods). Undoubtedly, the biggest advantage of VGA is its (partial) ability to treat tunneling at low temperature, which we demonstrated for some choices of double-well potential parameters. Further, it produces *coherent* tunneling, as shown in Figure 1. No other method (RPMD, CW, PILD, PFK-LPI, etc.) is anywhere near this when applied to double-well potentials; see, e.g., Refs. [24,26].

Finally, we comment on the computational challenge of evaluating the multi- dimensional Gaussian-averaged potential and its second derivative, needed in the VGA dynamics; see Appendix A. A similar Gaussian integral of the Hessian of the potential arises in, e.g., the Feynman–Kleinert implementation of the classical Wigner model [28,29]. Here, the problem was solved by adopting an integration by parts of the integral, which turns the problem into one of averaging the potential *gradient* instead, which was performed via Monte Carlo sampling. In this way, arbitrary complex potentials can be studied. In Ref. [29], a 300-atom graphite surface with a complex non-pairwise potential was treated in this way, and in Ref. [28], two other applications can be found.

## Data Availability

The raw data supporting the conclusions of this article will be made available by the authors on request.

## Appendix A

The VGA wave packet can be written in the form

(A1) $$\langle x\left|g(x_{0},p_{0};t)\right.\rangle =\exp\left(\frac{1}{2}\frac{i}{\hbar }D\left(t\right){\left(x-x_{0}\left(t\right)\right)}^{2}+\frac{i}{\hbar }p_{0}\left(t\right)\left(x-x_{0}\left(t\right)\right)+\frac{i}{\hbar }w\left(t\right)\right),$$

where both $D\left(t\right)=D_{r}\left(t\right)+iD_{i}\left(t\right)$ and $w\left(t\right)=w_{r}\left(t\right)+iw_{i}\left(t\right)$ are complex. In Equation (A1), we prefer the factor $\frac{1}{2}\frac{i}{\hbar }D\left(t\right)$ instead of $\frac{i}{\hbar }D\left(t\right)$ as used in Refs. [2,6]. With this small modification in mind, the equations of motions can be found from [2,6]:

(A2) $${\dot{x}}_{0}=p_{0}/m;\;{\dot{p}}_{0}=-\langle \hat{V^{\prime }}\rangle ,$$

(A3) $$\dot{D}=-D{\left(t\right)}^{2}/m-\langle \hat{V^{\prime \prime }}\rangle ,$$

(A4) $$\dot{w}=\frac{p_{0}^{2}}{2m}+i\frac{\hbar }{2m}D-\langle \hat{V}\rangle +\frac{\hbar }{4D_{i}}\langle \hat{V^{\prime \prime }}\rangle ,$$

where $\langle \hat{\Omega }\rangle$ means the expectation value of $\hat{\Omega }$ using the VGA wavepacket in Equation (A1). In this work, we prefer the slightly different expression:

(A5) $$\langle x\left|g(x_{0},p_{0};t)\right.\rangle ={\left(\frac{A_{r}\left(t\right)}{\pi }\right)}^{\frac{1}{4}}\exp\left(-\frac{1}{2}A\left(t\right){\left(x-x_{0}\left(t\right)\right)}^{2}+\frac{i}{\hbar }p_{0}\left(t\right)\left(x-x_{0}\left(t\right)\right)+\frac{i}{\hbar }\gamma \left(t\right)\right),$$

where $A_{r}\left(t\right)$ is the real part of the complex width A(t) and γ(t) is real. The difference between these two expressions is that the imaginary part of w(t) has been removed and written explicitly as a normalization factor in Equation (A5). The equations of motion for A(t) and γ(t) are obtained easily. We notice that

(A6) $$-\frac{i}{\hbar }D\left(t\right)=A\left(t\right)\Leftrightarrow A_{r}=D_{i}/\hbar \;,\;A_{i}=-D_{r}/\hbar .$$

Then, Equation (A3) leads to

(A7) $$i\hbar \dot{A}=\hbar^{2}A{\left(t\right)}^{2}/m-\langle \hat{V^{\prime \prime }}\rangle .$$

The equation for γ is obtained by retaining only the real part of Equation (A4):

(A8) $$\dot{\gamma }=\frac{p_{0}^{2}}{2m}-\frac{\hbar^{2}}{2m}A_{r}-\langle \hat{V}\rangle +\frac{1}{4A_{r}}\langle \hat{V^{\prime \prime }}\rangle .$$

## Appendix B

Here, we derive Equation (15). First, we substitute Equation (4) into Equation (1) and obtain

$$G\left(t\right)=\frac{1}{Z}Tr\left\{\hat{x}\left(t\right)\exp\left(-\frac{\beta }{2}\hat{H}\right)\hat{x}\exp\left(-\frac{\beta }{2}\hat{H}\right)\right\}$$

(A9) $$\approx \frac{1}{Z}\int dx_{0}x_{0}Tr\left\{\exp\left(i\hat{H}t/\hbar \right)\hat{x}\exp\left(-i\hat{H}t/\hbar \right)|g(x_{0};\frac{\beta }{2})\rangle \langle g(x_{0};\frac{\beta }{2})|\right\}$$

$$=\frac{1}{Z}\int dx_{0}x_{0}Tr\left\{\hat{x}\exp\left(-i\hat{H}t/\hbar \right)|g(x_{0};\frac{\beta }{2})\rangle \langle g(x_{0};\frac{\beta }{2})|\exp\left(i\hat{H}t/\hbar \right)\right\}$$

$$=\frac{1}{Z}\int dx_{0}x_{0}Tr\left\{\hat{x}|g(x_{0};\frac{\beta }{2})\rangle \langle g(x_{0};\frac{\beta }{2})|\left(-t\right)\right\}$$

$$=\frac{1}{Z}\int dx_{0}x_{0}Tr\left\{\hat{x}|g(x_{0};\frac{\beta }{2})\rangle \langle g(x_{0};\frac{\beta }{2})|\left(t\right)\right\},$$

where the last equality follows, since G(t) is real; therefore, G(t)=G(−t). Next, we evaluate the trace in Wigner phase space by using

(A10) $$Tr\left\{\hat{A}\hat{B}\right\}=\int \int \frac{dqdp}{2\pi \hbar }{\left(\hat{A}\right)}_{W}\left[q,p\right]{\left(\hat{B}\right)}_{W}\left[q,p\right].$$

Then, Equation (A9) turns into

$$G\left(t\right)\approx \frac{1}{Z}\int dx_{0}x_{0}\int \int \frac{dqdp}{2\pi \hbar }{\left(\hat{x}\right)}_{W}\left[q,p\right]{\left(|g(x_{0};\frac{\beta }{2})\rangle \langle g(x_{0};\frac{\beta }{2})|\left(t\right)\right)}_{W}\left[q,p\right]$$

(A11) $$=\frac{1}{Z}\int dx_{0}x_{0}\int \int \frac{dqdp}{2\pi \hbar }q{\left(|g(x_{0};\frac{\beta }{2})\rangle \langle g(x_{0};\frac{\beta }{2})|\left(t\right)\right)}_{W}\left[q,p\right].$$

The thermal Wigner function ${\left(|g(x_{0};\frac{\beta }{2})\rangle \langle g(x_{0};\frac{\beta }{2})|\left(t\right)\right)}_{W}\left[q,p\right]$ is of the form given by Equation (10) and is propagated by using the VGA equations in Equation (11). At t=0, we may write it explicitly as $\alpha =\Lambda (\frac{\beta }{2})$ and $\lambda =1/(\hbar^{2}\Lambda \left(\frac{\beta }{2}\right))$:

$${\left(|g(x_{0};\frac{\beta }{2})\rangle \langle g(x_{0};\frac{\beta }{2})|\left(t=0\right)\right)}_{W}\left[q,p\right]$$

(A12) $$=2\exp\left(2\gamma \left(\frac{\beta }{2}\right)\right)\sqrt{\frac{\pi }{\Lambda (\frac{\beta }{2})}}\exp\left(-{\left(\begin{array}{c} q-x_{0}\left(\frac{\beta }{2}\right)\\ p \end{array}\right)}^{T}\left(\begin{array}{cc} \Lambda (\frac{\beta }{2}) & 0\\ 0 & 1/(\hbar^{2}\Lambda \left(\frac{\beta }{2}\right)) \end{array}\right)\left(\begin{array}{c} q-x_{0}\left(\frac{\beta }{2}\right)\\ p \end{array}\right)\right),$$

which follows from Equation (3). It is not normalized in Wigner phase space:

$$\int \int \frac{dqdp}{2\pi \hbar }{\left(|g(x_{0};\frac{\beta }{2})\rangle \langle g(x_{0};\frac{\beta }{2})|\left(t=0\right)\right)}_{W}\left[q,p\right]$$

(A13) $$=\exp\left(2\gamma \left(\frac{\beta }{2}\right)\right)\sqrt{\frac{\pi }{\Lambda (\frac{\beta }{2})}}.$$

The value of Equation (A13) is the same, even if t≠0, which follows from the fact that $\alpha \left(t\right)\lambda \left(t\right)-\theta {\left(t\right)}^{2}=1/\hbar^{2}$ for all *t* as mentioned after Equation (14). Thus, it follows that

$${\left(|g(x_{0};\frac{\beta }{2})\rangle \langle g(x_{0};\frac{\beta }{2})|\left(t\right)\right)}_{W}\left[q,p\right]=2\exp\left(2\gamma \left(\frac{\beta }{2}\right)\right)$$

(A14) $$\times \sqrt{\frac{\pi }{\Lambda (\frac{\beta }{2})}}\exp\left(-{\left(\begin{array}{c} q-q_{0}\left(t\right)\\ p-p_{0}\left(t\right) \end{array}\right)}^{T}\left(\begin{array}{cc} \alpha (t) & \theta (t)\\ \theta (t) & \lambda (t) \end{array}\right)\left(\begin{array}{c} q-q_{0}\left(t\right)\\ p-p_{0}\left(t\right) \end{array}\right)\right),$$

and thus that

$$\int \int \frac{dqdp}{2\pi \hbar }q{\left(|g(x_{0};\frac{\beta }{2})\rangle \langle g(x_{0};\frac{\beta }{2})|\left(t\right)\right)}_{W}\left[q,p\right]$$

(A15) $$=\exp\left(2\gamma \left(\frac{\beta }{2}\right)\right)\sqrt{\frac{\pi }{\Lambda (\frac{\beta }{2})}}\times q_{0}\left(t\right).$$

Inserting this result into Equation (A11) yields

(A16) $$G\left(t\right)\approx \frac{1}{Z}\int dx_{0}x_{0}\exp\left(2\gamma \left(\frac{\beta }{2}\right)\right)\sqrt{\frac{\pi }{\Lambda (\frac{\beta }{2})}}\times q_{0}\left(t\right),$$

which is Equation (15).

## Appendix C

We use the RPMD algorithm to calculate an approximation to the Kubo-transformed position auto-correlation function, which is given by

(A17) $$C_{K}\left(t\right)=\frac{1}{\beta \hbar Z}\int_{0}^{\beta \hbar }d\lambda Tr\left\{\exp\left(i\hat{H}t/\hbar \right)\hat{x}\exp\left(-i\hat{H}t/\hbar \right)\exp\left(-\left(\beta -\lambda \right)H\right)\hat{x}\exp\left(-\lambda \hat{H}\right)\right\}.$$

Here, we show how to calculate G(t), defined in Equation (1), from $C_{K}\left(t\right)$. By using an energy eigenbasis of Equations (1) and (A17), we obtain the Fourier relation

(A18) $$G\left(\omega \right)=\frac{\beta \hbar \omega /2}{\sinh(\beta \hbar \omega /2)}C_{K}\left(\omega \right).$$

We may, therefore, write

$$G\left(t\right)=\frac{1}{2\pi }\int_{-\infty }^{\infty }d\omega \exp\left(i\omega t\right)\frac{\beta \hbar \omega /2}{\sinh(\beta \hbar \omega /2)}\int_{-\infty }^{\infty }ds\exp\left(-i\omega s\right)C_{K}\left(s\right)$$

(A19) $$=\frac{1}{2\pi }\int_{-\infty }^{\infty }ds\int_{-\infty }^{\infty }d\omega \frac{\beta \hbar \omega /2}{\sinh(\beta \hbar \omega /2)}\exp\left(-i\omega \left(s-t\right)\right)C_{K}\left(s\right)$$

$$=\frac{1}{2\pi }\int_{-\infty }^{\infty }dsK\left(s-t\right)C_{K}\left(s\right),$$

with

(A20) $$K\left(s-t\right)=\int_{-\infty }^{\infty }d\omega \frac{\beta \hbar \omega /2}{\sinh(\beta \hbar \omega /2)}\exp\left(-i\omega \left(s-t\right)\right)$$

$$=\frac{2}{\beta \hbar }\int_{-\infty }^{\infty }dy\frac{y}{\sinh(y)}\cos\left(\frac{2y}{\beta \hbar }\left(s-t\right)\right),$$

where we have put y=βℏω/2. By using the result

(A21) $$\int_{-\infty }^{\infty }\frac{x}{\sinh(x)}\cos\left(ax\right)dx=\frac{\pi^{2}}{1+\cosh(\pi a)},$$

we obtain

(A22) $$K\left(s-t\right)=\frac{2}{\beta \hbar }\frac{\pi^{2}}{1+\cosh(\pi \frac{2}{\beta \hbar }\left(s-t\right))}.$$

Substituting this result into Equation (A19) leads to

$$G\left(t\right)=\frac{1}{2\pi }\int_{-\infty }^{\infty }dsK\left(s-t\right)C_{K}\left(s\right)$$

(A23) $$=\frac{\pi }{\beta \hbar }\int_{-\infty }^{\infty }ds\frac{C_{K}\left(s\right)}{1+\cosh(\pi \frac{2}{\beta \hbar }\left(s-t\right))}.$$

The integrand in this equation that multiplies $C_{K}\left(s\right)$ is a strongly peaked function centered around s=t with a width approximately equal to βℏ/2π. Hence, at high temperature, G(t) becomes equal to $C_{K}\left(t\right)$.
